# Internalized stigma, depression and quality of life in schizophrenia

**DOI:** 10.1192/j.eurpsy.2021.1374

**Published:** 2021-08-13

**Authors:** S. Elleuch, N. Smaoui, R. Feki, M. Maalej Bouali, S. Omri, N. Charfi, J. Ben Thabet, L. Zouari, M. Maalej

**Affiliations:** Psychiatry C Department, Hedi chaker University hospital, sfax, Tunisia

**Keywords:** internal stigma, schizophrénia, Depression, quality of life

## Abstract

**Introduction:**

People with a schizophrenia experience higher levels of stigma.

**Objectives:**

Our aim was to explore the relationship between internalized stigma, depression and quality of life (QoL) in these patients.

**Methods:**

This is a cross-sectional and analytical study including 37 stabilized patients with schizophrenia or schizoaffective disorder followed up in the outpatient psychiatry department at Hedi Chaker hospital university of Sfax, between August and October 2019. The Internalized Stigma of Mental Illness scale (ISMI-29) was used to assess internalized stigma and its five dimensions. We used the Quality of Life Enjoyment and Satisfaction Questionnaire (Q-LES-Q-SF) to assess QoL and the Calgary Depression Scale (CDS) to evaluate depression.

**Results:**

73% of these patients were followed for schizophrenia and 27% for schizoaffective disorder. The global mean score of ISMI was 71.95. The mean scores of alienation, stereotype endorsement, perceived discrimination, social withdrawal and stigma resistance were 15.16, 16.54, 12.95, 15.65 and 11.38, respectively. The Q-LES-Q-SF mean score was 65.51. According to CDS, 18.9% of patients had depression with a mean score of 2.27. Internalized stigma scores (global and the five dimensions scores) were significantly and negatively associated with QoL enjoyment satisfaction score (respective p: p<0.001;p<0.001; p=0.004; p<0.001; p<0.001; p<0,001; p<0.001). Global ISMI score and the four first dimensions scores were positively associated with depressive patients (respective p: p=0.002, p<0.001, p=0.025, p=0.001 and p=0.003) while stigma resistance was negatively correlated with depression (p<0.001).

**Conclusions:**

Our results confirmed that internalized stigma is associated with impaired QoL and depression in stabilized patients with schizophrenia-spectrum diagnosis.

